# Treating Pulmonary Arterial Hypertension With Sotatercept: A Meta-Analysis

**DOI:** 10.7759/cureus.51867

**Published:** 2024-01-08

**Authors:** Naseer Uddin, Muhammad Talal Ashraf, Stafford Jude Sam, Affan Sohail, Syed Muhammad Ismail, Antonella Paladini, Abdul Ahad Syed, Tamam Mohamad, Giustino Varrassi, Satish Kumar, Mahima Khatri

**Affiliations:** 1 Department of Medicine, Dow University of Health Sciences, Civil Hospital Karachi, Karachi, PAK; 2 Department of Internal Medicine/Cardiology, Dow University of Health Sciences, Civil Hospital Karachi, Karachi, PAK; 3 Department of MESVA, University of L'Aquila, L'Aquila, ITA; 4 Department of Cardiovascular Medicine, Wayne State University, Detroit, USA; 5 Department of Pain Medicine, Paolo Procacci Foundation, Rome, ITA; 6 Department of Medicine, Shaheed Mohtarma Benazir Bhutto Medical College, Karachi, PAK; 7 Department of Internal Medicine/Cardiology, Dow University of Health Sciences, Karachi, PAK

**Keywords:** meta-analysis, pulmonary arterial hypertension, pulmonology, drug therapy, sotatercept

## Abstract

Pulmonary arterial hypertension (PAH) results from proliferative remodeling and narrowing of the pulmonary vasculature. Sotatercept is a first-in-class fusion protein that has recently garnered attention for showing improvements in patients with PAH. This meta-analysis of randomized controlled trials (RCTs) assesses the overall efficacy of Sotatercept in treating PAH.

PubMed, Google Scholar, and Clinicaltrials.gov were searched using relevant keywords and MeSH terms. Studies were included if RCTs compared Sotatercept with placebo in patients with PAH.

Our comprehensive literature search yielded 3,127 results, of which two RCTs with 429 patients were included in this meta-analysis. The patients were on background therapy for PAH. Results of the meta-analysis show that when compared with placebo, Sotatercept improved the six-minute walk distance (mean difference [MD] 34.99; 95% confidence interval [CI] 19.02-50.95; *P *< 0.0001), the World Health Organization (WHO) functional class (odds ratio [OR] 2.50; 95% CI 1.50-4.15; *P *= 0.0004), and pulmonary vascular resistance (PVR, MD -253.90; 95% CI -356.05 to -151.75; *P *< 0.00001). However, reduction in N-terminal pro-B-type natriuretic peptide (NT-proBNP, MD -1563.14; 95% CI -3271.93 to 145.65; *P *= 0.07) was not statistically significant in the Sotatercept group versus placebo.

In conclusion, Sotatercept improves the six-minute walk distance, WHO functional class, and PVR in patients with PAH receiving background therapy. However, the effect on NT-proBNP levels was not statistically significant. More research is needed to assess the clinical relevance of these findings.

## Introduction and background

Pulmonary arterial hypertension (PAH) is the increased pulmonary arterial pressure resulting from the proliferative remodeling and narrowing of the pulmonary vasculature [1]. One percent of the world's population is affected by PAH, with a notable prevalence among older individuals. Consequently, the burden of this condition is quite significant [2]. PAH can also lead to right-sided heart failure and death. PAH is managed using phosphodiesterase-5 inhibitors, guanylate cyclase stimulators, endothelin-receptor antagonists, and drugs interfering with the prostacyclin pathway [2,3,4]. However, there has not been improvement in the survival of patients, which expresses the need for new treatment options targeting novel ways involved in the pathogenesis of PAH [5]. The vascular remodeling in PAH is primarily driven by increased proliferation in the endothelial and muscular layers and decreased apoptosis [6]. Research has unraveled the role of transforming growth factor β (TGF-β) super-family members, including activin receptor type IIA (ActRIIA), ActRIIA ligands activin A, activin B, and others in the development of PAH [7-9]. Sotatercept is a first-in-class fusion protein that acts as a ligand trap for selected TGF-β superfamily members involved in the pathophysiology of PAH [10,11]. Data on the efficacy of Sotatercept for treating PAH is limited. This meta-analysis aims to assess the overall effectiveness of Sotatercept in treating PAH from the available clinical trials.

## Review

Methodology

Preferred Reporting Items for Systematic Reviews and Meta-Analyses (PRISMA) guidelines were followed while conducting this meta-analysis [12].

Search and Study Selection

PubMed, Google Scholar, and Clinicaltrials.gov were searched from inception to June 8, 2023, using the keywords and MeSH terms: "Sotatercept," "ACE-011," "ActRIIA-IgG1," "ACTRIIA-Fc," "MK-7962," "RAP-011," "ActRIIA-IgG1Fc," "Activin receptor type IIA antagonist," and "Pulmonary hypertension." Studies were selected if they were (1) RCTs and (2) included patients with PAH randomized between Sotatercept and placebo. PubMed-suggested articles and reference lists of included articles were searched to find related studies.

Outcomes

Outcomes of interest for this meta-analysis included change from baseline in a six-minute walk distance, the World Health Organization (WHO) functional class improvement, pulmonary vascular resistance (PVR), and N-terminal pro-B-type natriuretic peptide (NT-proBNP).

Data Extraction

Data extracted from the studies included study and patient characteristics (author name, study design, phase of study, duration of study, sample size, mean age, number of female participants, classification of PAH, background therapy, WHO functional class, six-minute walk distance, PVR, and NT-proBNP). Outcome data were extracted for the six-minute walk distance, WHO operational class improvement, PVR, and NT-proBNP.

Statistical Analysis

RevMan V.5.3 (Cochrane Collaboration) software was used for the meta-analysis of data [13]. Mean change, confidence interval (CI), standard error (SE), and sample size were extracted for continuous outcomes. In contrast, for dichotomous outcomes, the number of events in each group and their sample sizes were extracted and pooled using the random effects model to obtain the summary statistics [14]. A *P*-value of <0.05 was considered significant. Higgins' I2 was used to assess heterogeneity, and the I2 value >50% was considered important [15-17].

Results

Study Selection

**Figure 1:**
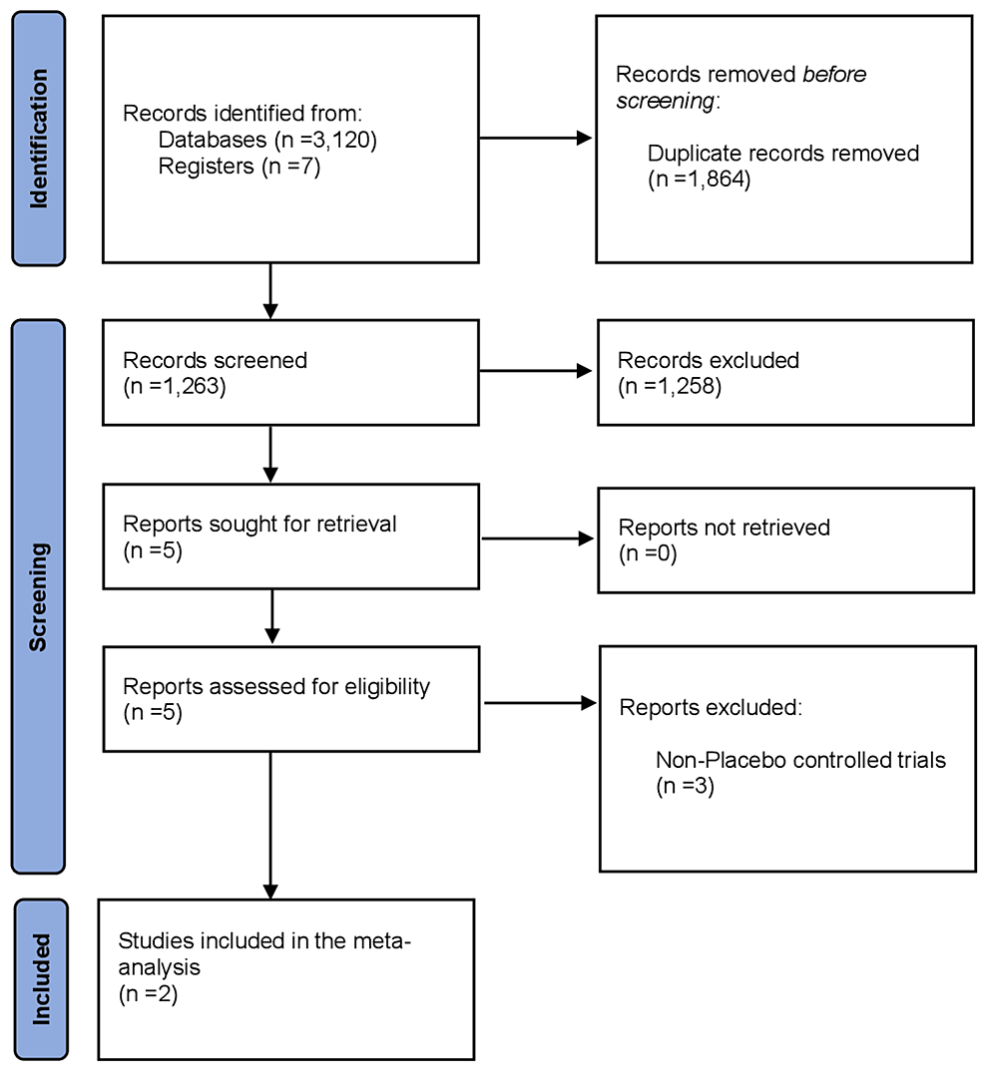
PRISMA flow diagram showing the study selection process. PRISMA, Preferred Reporting Items for Systematic Reviews and Meta-Analyses

The initial search yielded 3,127 results. A total of 1,864 were removed as duplicates, 1,258 were excluded during the screening process, and three more were excluded due to nonrandomized study design. In the end, two RCTs involving 429 patients with PAH were included in this meta-analysis. Patients were on background therapy for PAH in both trials [18,19]. PRISMA flow diagram is shown in Figure 1.

Study and Patient Characteristics of the Included Trials

We included phase II and III trials. Among 429 patients, 237 were given Sotatercept and 192 were given the placebo. The mean age of the included patients was 48.55 and 46.95 years in the Sotatercept and placebo groups, respectively. The study and patient characteristics of the included trials are given in Table 1. 

**Table 1:** Study and patient characteristics of the included trials. N/A, not available

Author and study name, year of publication	Study design	Phase of study	Duration of study - weeks (timeline)	Sample size (*n*)	Mean age (years)	Females, *n* (%)	Classification of pulmonary arterial hypertension, *n* (%)			Background therapy, *n* (%)			WHO functional class, *n* (%)			Six-minute walk distance (m)	Pulmonary vascular resistance (dyn.second.cm^-5^)	NT-proBNP (pg/mL)
Hoeper et al. (2021) [18]	Randomized controlled trial	3	24 (January 2021 to August 2022)	Sotatercept = 163 Placebo = 160	Sotatercept =47.6 ± 14.1 Placebo = 48.3 ± 15.5	Sotatercept = 129 (79.1) Placebo = 127 (79.4)	Idiopathic	Sotatercept = 83 (50.9) Placebo = 106 (66.2)		Prostacyclin Infusion therapy	Sotatercept = 65 (39.9) Placebo = 64 (40.0)		II	Sotatercept = 79 (48.5) Placebo = 78 (48.8)		Sotatercept = 397.6 ± 84.3 Placebo = 404.7 ± 80.6	Sotatercept = 781.3 ± 398.5 Placebo = 745.8 ± 313.5	Sotatercept =1037.5 ± 2498.6 Placebo = 1207.8 ± 2694.4
							Heritable	Sotatercept = 35 (21.5) Placebo = 24 (15.0)					III	Sotatercept = 84 (51.5) Placebo = 82 (51.2)				
							Associated with connective- tissue disease	Sotatercept = 29 (17.8) Placebo = 19 (11.9)		Monotherapy	Sotatercept = 9 (5.5) Placebo = 4 (2.5)		N/A	N/A				
							Drug-induced or toxin-induced	Sotatercept = 7 (4.3) Placebo = 4 (2.5)		Double therapy	Sotatercept = 56 (34.4) Placebo = 56 (35.0)		N/A	N/A				
							Associated with corrected congenital shunts		Sotatercept = 9 (5.5) Placebo = 7 (4.4)	Triple therapy		Sotatercept = 98 (60.1) Placebo = 100 (62.5)	N/A	N/A				
Humbert et al. (2023) [19]	Randomized controlled trial	2	24 (June 2018 to December 2019)	Sotatercept = 74 Placebo = 32	Sotatercept = 49.5 ± 14.7 Placebo = 45.6 ± 13.4	Sotatercept = 66 (89) Placebo = 26 (81)	Idiopathic			Prostacyclin Infusion therapy		Sotatercept = 29 (39) Placebo = 10 (31)	II		Sotatercept = 39 (53) Placebo = 17 (53)	Sotatercept = 392.5 ± 89.9 Placebo = 409.1 ± 63.9	Sotatercept = 770.4 ± 361.0 Placebo = 797.4 ± 322.6	Sotatercept = 924.9 ± 1465.2 Placebo = 870.2 ± 1213.3
							Heritable		Sotatercept = 10 (14) Placebo = 7 (22)				III		Sotatercept = 35 (47) Placebo = 15 (47)			
							Associated with connective- tissue disease		Sotatercept = 15 (20) Placebo = 3 (9)	Monotherapy		Sotatercept = 7 (9) Placebo = 3 (9)			N/A			
							Drug-induced or toxin-induced		Sotatercept = 6 (8) Placebo = 1 (3)	Double therapy		Sotatercept = 25 (34) Placebo = 12 (38)			N/A			
							Associated with corrected congenital shunts		Sotatercept = 1 (1) Placebo = 2 (6)	Triple therapy		Sotatercept = 42 (57) Placebo = 17 (53)			N/A			

Outcomes

**Figure 2:**
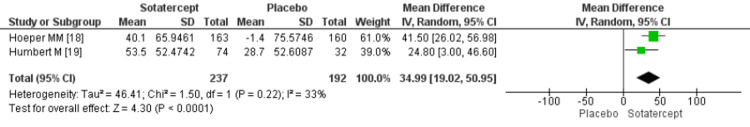
Forest plot of change in a six-minute walk distance. CI, confidence interval, SD, standard deviation; IV, intravenous

Six-minute walk distance: Pooled analysis showed that Sotatercept significantly improved the six-minute walk distance compared to placebo (MD 34.99; 95% CI 19.02-50.95; *P *< 0.0001; heterogeneity *P *= 0.22; I2 = 33%), as shown in Figure 2.

**Figure 3:**
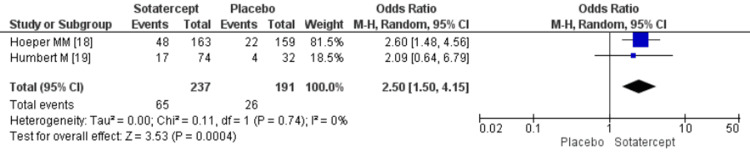
Forest plot of WHO functional class improvement. CI, confidence interval; WHO, World Health Organization

WHO functional class: Sotatercept also improved the WHO functional class in the treatment group compared to placebo (odds ratio [OR] 2.50; 95% CI 1.50-4.15; *P*=0.0004; heterogeneity *P *= 0.74; I2 = 0%), as shown in Figure 3.

**Figure 4:**
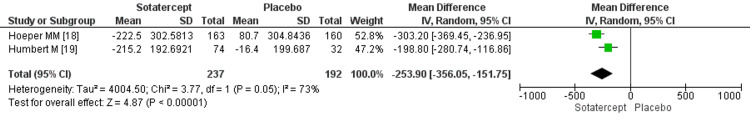
Forest plot of change in pulmonary vascular resistance. CI, confidence interval, SD, standard deviation

Pulmonary vascular resistance: PVR was also improved in the Sotatercept treatment group compared to placebo (MD -253.90; 95% CI -356.05 to -151.75; *P *< 0.00001; heterogeneity *P *= 0.05; I2 = 73%), as shown in Figure 4.

**Figure 5:**
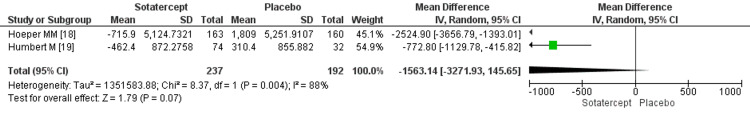
Forest plot of change in NT-proBNP. CI, confidence interval, SD, standard deviation; NT-proBNP, N-terminal pro-B-type natriuretic peptide

NT-proBNP: Treatment with Sotatercept did not show a statistically significant reduction in NT-proBNP (MD -1563.14; 95% CI -3271.93 to 145.65; *P *= 0.07; heterogeneity *P *= 0.004; I2 = 88%) in the treatment group compared to placebo, as shown in Figure 5.

Discussion

This meta-analysis evaluated the efficacy of Sotatercept in treating PAH by analyzing the results of two RCTs involving 429 patients [18,19]. The results showed several benefits of Sotatercept, including improvements in the six-minute walk distance, WHO functional class, and PVR. The analysis revealed a statistically significant increase in the six-minute walk distance in the Sotatercept group compared to the placebo group, indicating that Sotatercept improves exercise capacity in PAH patients. Furthermore, the analysis showed a significant improvement in WHO functional class in the Sotatercept group, a reduction in symptoms, and functional limitations in patients with PAH. A substantial decrease in the hemodynamic parameter of PVR was also observed with Sotatercept treatment, indicating that the drug may directly alter the remodeling process of the vasculature [10,20]. PVR is an important prognostic factor in PAH, and it predicts hospitalization for heart failure and mortality [21]. However, regarding the reduction in NT-proBNP levels, the difference between the Sotatercept and placebo groups was not statistically significant. Side effects of treatment include bleeding, mainly epistaxis, gingival bleeding, and telangiectasia. An increase in hemoglobin level is also seen as a side effect of the treatment [18,19]. Around 33% of the PAH patients are anemic, and both anemia and polycythemia negatively affect the prognosis of the disease [22,23]. Future studies may investigate the erythropoietic effect of Sotatercept and its influence on clinical outcomes. These outcomes were not included in the results of interest of this study and hence were not analyzed.

Immunogenicity and Pharmacokinetics of Sotatercept: Understanding Its Impact and Future Implications

Introducing biologics, such as Sotatercept, has dramatically enhanced the range of medical therapeutic interventions. Sotatercept is a genetically engineered fusion protein that targets explicitly critical processes implicated in the development of diseases [24]. It is crucial to prioritize the study of the immunogenicity and pharmacokinetics of these biologics to enhance their effectiveness and ensure their safety. Immunogenicity, which pertains to the capacity of a drug to elicit an immune response, is a crucial determinant impacting the pharmacokinetics, and hence, the therapeutic effectiveness and safety of biologic medicines such as Sotatercept [24]. Sotatercept, as a fusion protein, is intended to regulate specific biological pathways. Nevertheless, when foreign proteins are introduced into the human body, they might trigger an immunological response, creating antidrug antibodies (ADAs). ADAs can have a profound impact on the pharmacokinetics of Sotatercept. This modification primarily appears to alter the drug's distribution, metabolism, and excretion mechanisms, affecting its overall therapeutic effectiveness [5]. For example, the development of ADAs might result in a faster elimination of the medication from the body, causing a decrease in its concentration in the blood and, as a result, limiting its therapeutic efficacy.

Furthermore, the immunogenicity of Sotatercept differs between individuals due to innate variations in immune system functionality and genetic predispositions. The heterogeneity in patient responses might result in a broad spectrum of outcomes, ranging from the desired therapeutic effect to reduced effectiveness or adverse immunological reactions. These variances highlight the need for individualized treatment methods while delivering Sotatercept [6]. Personalized medicine, in this sense, refers to customizing the pharmacological treatment plan according to specific patient attributes, including genetic composition, immune system condition, and preexisting ADAs. This method aims to optimize effectiveness while minimizing possible negative consequences [7]. Moreover, how Sotatercept interacts with the immune system has broader implications for its prolonged usage and the advancement of comparable biological agents. Comprehending the elements that impact the ability to provoke an immune response, such as the arrangement of molecules, the schedule of doses, and the method of administration, is essential for enhancing the development and distribution of these treatments [11]. Regular monitoring and evaluation of immune responses in patients undergoing Sotatercept treatment are crucial for improving treatment regimens and optimizing patient outcomes.

Consequently, the future application of Sotatercept depends on a thorough comprehension of its immunogenic profile and pharmacokinetic properties. Research should focus on developing biomarkers to predict immunogenic responses and inform personalized therapy regimens accurately [24]. Moreover, progress in biotechnology may facilitate the development of biological medicines with less Immunogenicity, thereby improving their safety and effectiveness. The relationship between the immunogenicity of Sotatercept and its pharmacokinetics and efficacy is a multifaceted interaction that requires an individualized therapeutic approach [25]. The diversity in patient reactions emphasizes the necessity for customized treatment plans specifically designed to match the distinct immunogenic characteristics of each patient. Examining these facets has significant ramifications for the clinical application of Sotatercept and steers the forthcoming advancement of biological treatments [11]. As our comprehension of these mechanisms progresses, our capacity to efficiently employ these potent therapeutic agents in clinical settings will also advance, heralding a new era of precision medicine.

Hemodynamics and Right Heart Function

The study conducted by Souza et al. provides significant insights into the effects of Sotatercept on the circulation of blood and the functioning of the right side of the heart, specifically about PAH. Sotatercept, a new medicinal compound, has been studied to determine its ability to regulate the intricate pathophysiology of PAH [25]. The study's findings are crucial in understanding how Sotatercept can modify the hemodynamic conditions in PAH and, therefore, impact proper heart function. PAH is defined by increased PVR, which results in more significant stress on the right ventricle (RV). This increased resistance to blood flow places excessive pressure on the RV, frequently leading to right heart failure, a significant contributor to death in individuals with PAH [25]. The work conducted by Souza et al. has demonstrated that Sotatercept effectively induces hemodynamic changes, which hold great potential in addressing fundamental pathophysiological problems.

The study emphasizes a notable decrease in PVR after the delivery of Sotatercept. The reduction in resistance is crucial since it directly relieves the burden on the RV, potentially enhancing proper heart function and patient prognosis [23]. Furthermore, the observed decline in mean pulmonary arterial pressure (mPAP) is another advantageous outcome of Sotatercept, which alleviates the right heart's workload. Moreover, the study highlights enhancements in cardiac output and proper ventricular ejection fraction, suggesting a beneficial influence on the performance of the right side of the heart [11]. These enhancements are vital, as they indicate that Sotatercept reduces PVR and improves the right heart's functioning ability. This dual effect is significant in the setting of PAH, where the occurrence of right heart failure continues to be a problematic consequence [9].

The findings of Souza et al. further enhance our comprehension of the role of Sotatercept in the pathophysiology of PAH. Sotatercept appears to target the fundamental causes of the disease rather than merely alleviating its symptoms by effectively decreasing PVR and enhancing proper heart function. This activity is particularly pertinent considering the intricate nature of PAH, which encompasses the remodeling of pulmonary blood vessels and the interaction of several cytokines and growth factors [13]. The study also elucidates the potential of Sotatercept as a disease-modifying drug in PAH. Unlike most existing treatments that primarily focus on alleviating symptoms, Sotatercept's capacity to enhance hemodynamics and improve proper heart function indicates a more profound and essential role in modifying the progression of the disease [15]. Overall, the research conducted by Souza et al. offers significant knowledge regarding the effects of Sotatercept on the circulatory system and the functioning of the right side of the heart in individuals with PAH. The observed enhancements in PVR, mPAP, and proper ventricular function are encouraging and indicate a substantial advancement in the treatment of PAH [18]. These findings not only improve the comprehension of Sotatercept's therapeutic function but also create opportunities for additional investigation into targeted therapies that tackle the underlying causes of PAH, thereby enhancing patient outcomes in this complex disease [23].

Safety Profile and Side Effects

An essential component of evaluating Sotatercept's clinical usefulness, particularly in treating disorders such as PAH, is examining its safety profile and potential adverse effects. The safety management of Sotatercept, a recombinant fusion protein, is particularly challenging due to its method of action, which directly affects the production of red blood cells and the formation of new blood vessels [25]. This paper centers on the safety data about Sotatercept, with a particular emphasis on the occurrence and handling of significant adverse effects such as bleeding and elevated hemoglobin levels. It also delves into the clinical considerations required to balance the drug's effectiveness and safety [17]. Using Sotatercept can lead to bleeding problems, a significant worry because of its potential effects on the vascular system. Due to its modulation of the TGF-β pathway, Sotatercept carries the potential to interfere with normal vascular processes, which are crucial for maintaining vascular integrity and facilitating repair [12]. Clinical trials and post-marketing surveillance have documented instances of bleeding, varying from minor mucosal bleeding to more substantial occurrences. Managing these instances of bleeding requires diligent patient monitoring, particularly for individuals with a documented bleeding condition or who are concurrently taking anticoagulant medications. Swift acknowledgment and response are crucial in minimizing severe effects [24].

Another notable consequence linked to the Sotatercept is the elevation of hemoglobin levels. Due to its impact on the production of red blood cells, Sotatercept might cause increased hemoglobin and hematocrit levels. This could result in health hazards such as thromboembolism and hypertension [25]. Regular monitoring of blood counts and proactive management techniques, such as dose modifications or temporary withdrawal of therapy, are necessary to address the occurrence of substantial erythrocytosis. The significance of Sotatercept's safety profile highlights the necessity of customized treatment strategies based on the patient's reaction and ability to tolerate the medication [2]. To effectively balance the effectiveness and safety of Sotatercept, it is necessary to have a detailed grasp of its pharmacodynamics and consider individual patient characteristics. Medical professionals must carefully consider the therapeutic advantages of Sotatercept, including its capacity to enhance hemodynamics and alleviate the impact of PAH, compared to the potential hazards posed by its side effects [4]. Adopting a comprehensive approach that encompasses patient education, consistent monitoring for adverse outcomes, and a willingness to modify treatment based on the patient's response is necessary to achieve this balance [4].

Furthermore, the careful selection of patients based on specific criteria and the assessment of their initial risk are crucial to maximize the safety of Sotatercept. Patients who have preexisting disorders that could worsen the risk of bleeding or blood clotting need to be carefully evaluated and may need different treatment approaches. Moreover, the enduring safety characteristics of Sotatercept are now being investigated, requiring constant monitoring and reporting of negative occurrences to improve its application in clinical settings [10]. The safety characteristics of Sotatercept, characterized by potential bleeding risks and elevated hemoglobin levels, necessitate meticulous oversight and cautious clinical assessment [20]. The optimal use of Sotatercept in therapy requires carefully balancing its effectiveness and safety. This necessitates a thorough strategy considering individual patient factors, regular monitoring, and flexible treatment regimens. As the clinical expertise with Sotatercept grows, so will the comprehension of its ideal utilization in managing intricate conditions such as PAH, emphasizing maximizing patient advantage while minimizing potential dangers [21].

Study Limitations

The limitations of this study are the short treatment duration of 24 weeks for both trials, enrollment of only specific WHO functional classes of PAH patients, lower numbers of patients with PAH associated with connective-tissue disease, congenital heart disease, drugs, toxins, as well as the minority groups, non-American and non-European patients.

## Conclusions

In conclusion, the results of this meta-analysis show that Sotatercept improves the six-minute walk distance, WHO functional class, and pulmonary vascular resistance in PAH patients receiving background therapy. However, the effect on NT-proBNP levels is not statistically significant.

Despite promising results, the clinical relevance of these findings needs to be assessed in future clinical trials with large sample sizes, long-term treatment duration, and a diverse population of PAH patients.
